# Effects of Cerebellar tDCS on Inhibitory Control: Evidence from a Go/NoGo Task

**DOI:** 10.1007/s12311-020-01165-z

**Published:** 2020-07-14

**Authors:** Daniela Mannarelli, Caterina Pauletti, Alessia Petritis, Roberto Delle Chiaie, Antonio Currà, Carlo Trompetto, Francesco Fattapposta

**Affiliations:** 1 Department of Human Neuroscience, Sapienza University of Rome, Viale dell’Università 30, Rome, Italy; 2 Department of Medical-Surgical Sciences and Biotechnologies, A. Fiorini Hospital, Terracina, LT, Sapienza University of Rome, Polo Pontino, Latina, Italy; 3 Department of Neuroscience, Rehabilitation, Ophthalmology, Genetics, Maternal and Child Health, University of Genova, Genoa, Italy; 4 Department of Neuroscience, IRCCS Ospedale Policlinico San Martino, Genoa, Italy

**Keywords:** Cerebellum, Healthy subjects, Cognition, Go/NoGo, tDCS

## Abstract

Response inhibition as an executive function refers to the ability to suppress inappropriate but prepotent responses. Several brain regions have been implicated in the process underlying inhibitory control, including the cerebellum. The aim of the present study was to explore the role of the cerebellum in executive functioning, particularly in response inhibition. For this purpose, we transitorily inhibited cerebellar activity by means of cathodal tDCS and studied the effects of this inhibition on ERP components elicited during a Go/NoGo task in healthy subjects. Sixteen healthy subjects underwent a Go/NoGo task prior to and after cathodal and sham cerebellar tDCS in separate sessions. A reduction in N2-NoGo amplitude and a prolongation in N2-NoGo latency emerged after cathodal tDCS whereas no differences were detected after sham stimulation. Moreover, commission errors in NoGo trials were significantly higher after cathodal tDCS than at the basal evaluation. No differences emerged between performances in Go trials and those after sham stimulation. These data indicate that cerebellar inhibition following cathodal stimulation alters the ability to allocate attentional resources to stimuli containing conflict information and the inhibitory control. The cerebellum may regulate the attentional mechanisms of stimulus orientation and inhibitory control both directly, by making predictions of errors or behaviors related to errors, and indirectly, by controlling the functioning of the cerebral cortical areas involved in the perception of conflict signals and of the basal ganglia involved in the inhibitory control of movement.

## Introduction

Response inhibition refers to the ability to suppress inappropriate but prepotent responses [1, 2]. Several brain regions, including the ventrolateral and dorsolateral prefrontal cortices, the supplementary motor area (SMA) [3–6], the inferior frontal gyrus (IFG), and the anterior cingulate cortex (ACC), have been implicated in the process underlying response inhibition [7–9].

Previous studies have pointed to a possible role of the cerebellum in executive functioning, specifically in response inhibition. Indeed, cerebellar activation has been documented both by functional neuroimaging studies in healthy subjects during selective cognitive tasks designed to explore inhibitory control, such as the Stroop Color Word Test or the “Tower of London” test [10–12], and by clinical studies on patients with cerebellar lesions [13–15]. However, cerebellar activation during cognitive tasks does not necessarily indicate cognitive processing but may instead reflect subtle contaminations by task-related motor demands. Moreover, lesions in the cerebellum often do not occur in isolation, making conclusions about the specific effects of cerebellar lesions difficult. The cerebellar role in response inhibition consequently has yet to be fully understood [16].

In a recent study by our group, cerebellar inhibition induced by means of cathodal transcranial direct current stimulation (tDCS) in healthy subjects revealed the presence of an executive attentive dysfunction during the attention network test (ANT) that was specifically related to the ability to promote an adequate discrimination of stimuli containing conflict or error information [17].

Response inhibition may be studied by means of the event-related potential (ERP) technique and examining the brain electrophysiological responses during a Go/NoGo task. Typically, in a Go/NoGo task, participants rapidly respond to Go stimuli while withholding responses to NoGo stimuli. Many studies have suggested that processing of the Go stimulus, which is generally related to a motor response, is associated with the elicitation of two specific psychophysiological components: the N2 component (with maximal central peak) [18], which reflects the initial orienting to the stimulus [19], and the P3 component (with maximal parietal peak) [20–22], which is related to the attentive discrimination of the stimulus [23, 24]. Similarly, the NoGo stimulus is associated with the appearance of the N2 component (with greater frontal amplitude) [18, 25] and P3 component (with greater central amplitude) [20, 21, 26, 27]. In particular, the NoGo-N2 component is considered an earlier step of response inhibition that indicates the conflict between the internal representation of the Go response and the NoGo stimulus has been detected [27–29]. By contrast, the NoGo-P3 component may represent a later stage of response inhibition that reflects both the response evaluation and the cancelation of the planned response [30–33].

The aim of the present study was to more thoroughly explore the role of the cerebellum in executive functioning, particularly in response inhibition. For this purpose, we transitorily inhibited cerebellar activity by means of cathodal tDCS and studied the effects on ERP components elicited during a Go/NoGo task in healthy subjects.

Non-invasive brain stimulation techniques, such as tDCS, can be used to gain valuable insights into the neural mechanisms underlying cognitive processing. TDCS allows polarity-dependent stimulation, which results in differential cortical excitability changes [34]. These so-called after-effects are believed to be mediated by changes in membrane polarization thresholds via glutamatergic synapses and intracortical interneurons [35]. Although the after-effects of tDCS may differ between subjects depending on the tDCS intensity applied, the duration, the timing [36, 37], and the cognitive status of the subjects [38–42], cathodal tDCS is believed to reduce neuronal excitability and anodal tDCS to increase it [34, 43].

As regards cerebellar tDCS, several studies have demonstrated that anodal stimulation does not induce cognitive effects if applied to healthy subjects. Indeed, it has been reported that functions already adequately expressed in basic conditions cannot be enhanced further [44, 45]. Cathodal cerebellar stimulation can instead affect the execution of cognitive tasks, particularly by altering the processing of the acoustic stimulus, and result in a dysfunction of the attentive executive network [17, 44, 45]. As suggested by the results of these studies, the presence of cerebellar inhibition after cathodal tDCS may induce hyperactivity in brain areas [17, 45] following a reduction in the physiological cerebellar-brain inhibition tone (CBI) [46] in multiple brain areas. The hyperactivity in these cortical regions may, however, also be associated with uncoordinated functioning as well as with possible cognitive dysfunctions [17].

From an anatomo-functional point of view, the right cerebellar hemisphere is believed to play a prominent role in executive functions [49, 50] in view of the cerebro-cerebellar cross-connections and the important role played by the left frontal-parietal areas in the control of executive processes [47, 48]. It is for this reason that the right cerebellar hemisphere was chosen as a target for the application of tDCS in the present study.

On the basis of these assumptions, we hypothesized that cathodal tDCS over the right cerebellum reduces the amplitude of the N2 and P3 components elicited during a Go/NoGo task, particularly following the NoGo stimulus.

## Methods

### Subjects

Sixteen right-handed, healthy subjects (8 male, 8 female; mean age 25.7 ± 3.5 years; range 22–35 years) were enrolled in a double-blind, sham-controlled, crossover study. None of the subjects had a history of neurological or psychiatric disease or of head injury, and none reported consuming excessive amounts of alcohol or were taking any medication that affects the central nervous system. Written informed consent was obtained from all the subjects prior to the experiment. The study was approved by the Local Medical Ethics Committee.

### Procedure

Each subject received cathodal or sham tDCS stimulation during two randomized tDCS sessions performed at least 6 days apart. Both before and after tDCS stimulation, subjects reported their attention, fatigue, and perceived pain using a self-scored visual analogue scale [51] and underwent a psychophysiological evaluation.

### tDCS

None of the subjects or ERP investigators, with the exception of the investigator who applied the tDCS, was aware of whether cathodal or sham stimulation was being performed. The experimenter was present in the room at the beginning of each session so as to be able to start the tDCS system; she was not in the room during the stimulation phase, though subjects could call her and interrupt the tDCS procedure if any problem arose. During tDCS session, subjects were not engaged in any specific motor or cognitive task.

TDCS over the right cerebellar hemisphere was applied by means of two sponge electrodes (surface area = 25 cm^2^) moistened with a saline solution. One electrode was centered over the right cerebellar cortex, 1 cm below and 4 cm lateral to the inion (corresponding approximately to the projection of cerebellar lobule VII onto the scalp). The other electrode was positioned over the right deltoid muscle [52]. The onset and offset of all the interventions (cathodal and sham) entailed increasing and decreasing the current, respectively, in a ramp-like manner over 10 s, a method shown to achieve a good level of blinding between sessions [45, 53, 54]. The stimulation intensity was set at 2 mA and delivered over the cerebellum for 20 min using a battery-operated, constant current stimulator (BrainSTIM EMS Srl, Bologna, Italy), which is similar to the intensity adopted by Ferrucci et al. (2008) [52] and is considered to be well below the tissue damage threshold [53, 55, 56]. In the sham condition, pseudo-stimulation (110 uA over 15 ms every 550 ms) instead of the stimulation current was applied for 20 min [45].

### Psychophysiological Evaluation

The psychophysiological evaluation consisted of a Go/NoGo task and a simple reaction time task separated by a short interval. The two tasks were delivered in a randomized order between subjects and between sessions within subjects.

#### Go/NoGo Task

Two consecutive blocks of an unwarned equiprobable auditory Go/NoGo task were presented via circumaural headphones. Each block consisted of 150 tones (intensity, 60 dB SPL; duration, 50 ms; rise/fall time, 5 ms) presented with a fixed stimulus onset asynchrony (SOA) of 1100 ms and a fixed inter-stimulus interval (ISI) of 1040 ms. Half the tones were 1000 Hz and the other half were 1500 Hz; the tones were delivered in a randomized order. Subjects were required to button-press with their dominant hand in response to one of the tones (named Go stimulus), while they had to ignore the other (named NoGo stimulus); the Go tone frequency alternated between the blocks, and the frequency of the first block Go tone was counterbalanced between participants to avoid any consistent sequence effects between subjects. The task lasted about 10 min.

#### Simple Reaction Time Task

A simple reaction time task was performed as a control measure. The task consisted of two consecutive blocks of 75 acoustic tones (intensity, 60 dB SPL; duration, 50 ms; rise/fall time, 5 ms) with a fixed SOA of 1100 ms. Tones of 1000 or 1500 Hz were presented; only one type of stimulus was presented in each block. The order of the blocks was counterbalanced between participants. The subjects were instructed to push a button upon presentation of each stimulus (named Go stimulus). The task lasted about 5 min.

### EEG Recording

Subjects were seated in an anatomic chair in a faradized and light-attenuated room. The electrophysiological signals were recorded by means of a 21-channel cap with active electrodes at the F3, Fz, F4, C3, Cz, C4, P3, Pz, and P4 sites, according to the International 10–20 System, referred to linked mastoids and grounded at the forehead. The vertical electro-oculogram (VEOG) was recorded from above and below the left eye; a horizontal EOG (HEOG) was also performed using electrodes placed at the two external canthi. All inter-electrode impedances were kept below 3 kOhm. EEG signals and EOG were filtered using a 0.01–30 Hz. The data were digitized with an analogue/digital converter at a sampling rate of 1024 Hz and stored on a hard disk. A Mizar Sirius EEG-EP multifunctional system was used.

### ERPs Analysis

EEG data were clipped offline into epochs of 800 ms with a baseline correction of 100 ms before each stimulus. A first automatic procedure was used to reject trials containing drift deflection exceeding ± 100 μV in any channel including EOG, according to clinical guidelines [57]. A further offline analysis was performed to exclude ocular artifacts (eye movements/blinks), according to a standard algorithm [58] implemented in our analyzer software (ERPLAB Toolbox). Trials containing artifacts were eliminated by computing the cross-covariance between the single-trial EOG waveform and a 200-ms step function and rejecting trials on which the maximum covariance exceeded a ± 15 μV threshold. Lastly, the detection of artifacts was verified by visual inspection. Artifact rejection accounted for 5.9 ± 0.6/150 (3.9%) of the trials for the Go/NoGo task and 3.7 ± 1.7/75 (4.9%) for the simple RT task.

For each subject, all the artifact-free trials were averaged per stimulus (Go, NoGo) and filtered with a low-pass digital filter of 20 Hz. The mean number of trials included was 138.1 ± 3.5/150 (92.1%) for the Go/NoGo task (*cathodal*: 138.7 ± 3.5/150 (92.5%) total; 138.1 ± 3.7 for the pre-tDCS session (Go, 69.4 ± 1.7; NoGo, 68.8 ± 2.5), 139.3 ± 3.3 for the post-tDCS session (Go, 69.5 ± 2.3; NoGo, 69.8 ± 1.7); *sham*: 137.4 ± 3.5/150 (91.6%) total; 137.3 ± 3.8 for the pre-tDCS session (Go, 69 ± 2.6; NoGo, 68.3 ± 2.2), 137.6 ± 3.3 for the post-tDCS session (Go, 69.4 ± 2.3; NoGo, 68.2 ± 2.2)). For the simple RT task, the mean number of trials included was 71.3 ± 1.6/75 (95.1%) (*cathodal*: 71.3 ± 1.4/75 (95.1%) total; 71.6 ± 1.0 for pre-tDCS session, 71.2 ± 1.7 for post-tDCS session; *sham*: 71.3 ± 1.7/75 (95.1%) total; 71.1 ± 1.7 for pre-tDCS session, 71.4 ± 1.8 for post-tDCS session).

Scalp electrode activity was measured at all the electrode sites in which Fz, Cz, and Pz were analyzed. Fz, Cz, and Pz were chosen for the analyses because ERP responses are largest on the midline locations. As regards the Go/NoGo task, the N2 amplitude was measured as the mean voltage between 180 and 240 ms after Go and NoGo stimuli, while the N2 latency was calculated as the midpoint latency, i.e., the time point that divided the area under the curve into two equal halves. The P3 amplitudes were calculated as the mean voltage between 250 and 500 ms in the Go and NoGo responses, respectively. The P3 latencies were also calculated for each stimulus as the midpoint latency of the same temporal window.

As regards the simple reaction time task, the P3 amplitude, defined as the mean voltage between 250 and 500 ms, and the P3 latencies, defined as the midpoint latency of the same temporal window, were calculated as grand average of the Go stimuli in the two blocks.

Performance measures were also obtained by calculating the mean reaction times (RTs) of correct responses (correct responses ranged between 180 and 1000 ms) and the accuracy of the responses expressed as the absolute number of errors (commission errors to NoGo trials, omission responses to Go trials or Go RT< 180 > 1000 ms for Go/NoGo task; omission responses or RT < 180 > 1000 ms for the simple reaction time task) for both the simple reaction time task and the Go/NoGo task.

### Statistical Analysis

Data are expressed as the mean (± 1 standard deviation) for continuous variables and as proportions for categorical variables. The Shapiro-Wilk test was applied to assess the normal distribution of the data. All the psychophysiological data were normally distributed so that a parametric statistics was applied, with the exception of errors that were not normally distributed and required a non-parametric statistics.

For the Go/NoGo task, the ERP parameters (N2 and P3 amplitudes and latencies) were analyzed separately by means of ANOVA for repeated measures (rmANOVA), with the “stimulus” (Go-NoGo), the experimental “condition” (cathodal, sham), the “electrode” (Fz, Cz, Pz), and the “timing” (pre-tDCS and post-tDCS) as the within-subject factors. When necessary, a post hoc correction according to Bonferroni was then applied. Degrees of freedom were adjusted, when necessary, by using the Greenhouse-Geisser epsilon coefficient for possible violations of the sphericity assumption. Corrected *p* values are reported; the original degrees of freedom are reported together with their correction factor epsilon. Effect sizes were measured by calculating the partial eta squared (*η*^2^_p_). We interpreted the magnitude of the effect size provided by *η*^2^_p_ according to the following guidelines: = 0.01 small effect; = 0.06 medium effect; = 0.14 large effect [59].

Similarly, for the SRT task, ERP parameters (P3 amplitudes and latencies) were analyzed separately by means of rmANOVA, with the experimental “condition” (cathodal, sham), the “electrode” (Fz, Cz, Pz), and the “timing” (pre-tDCS and post-tDCS) as the within-subject factors. When necessary, a post hoc correction according to Bonferroni was then applied.

Incorrect responses (omission errors to Go trials in the Go/NoGo task and to SRT trials, and commission errors to NoGo trials or false alarms (FA)) were analyzed separately for each condition and timing (pre-cathodal, post-cathodal, pre-sham, post-sham) by means of Friedman test followed, when necessary, by the Wilcoxon signed-rank post hoc test with Bonferroni’s correction.

Finally, reaction times (RT) in the Go trials in the Go/NoGo task and those in the SRT trials were analyzed separately by means of rmANOVA, with the experimental “condition” (cathodal, sham) and the “timing” (pre-tDCS and post-tDCS) as the within-subject factors.

Attention, fatigue, and perceived pain pre-tDCS and post-tDCS were analyzed separately for each condition (cathodal, anodal, sham) by means of the Wilcoxon signed-rank test. A post hoc correction according to Bonferroni was applied if necessary.

The significance level was set at *p* ≤ 0.05. The analyses were performed using the SPSS statistical package (Version 25).

## Results

**Fig. 1:**
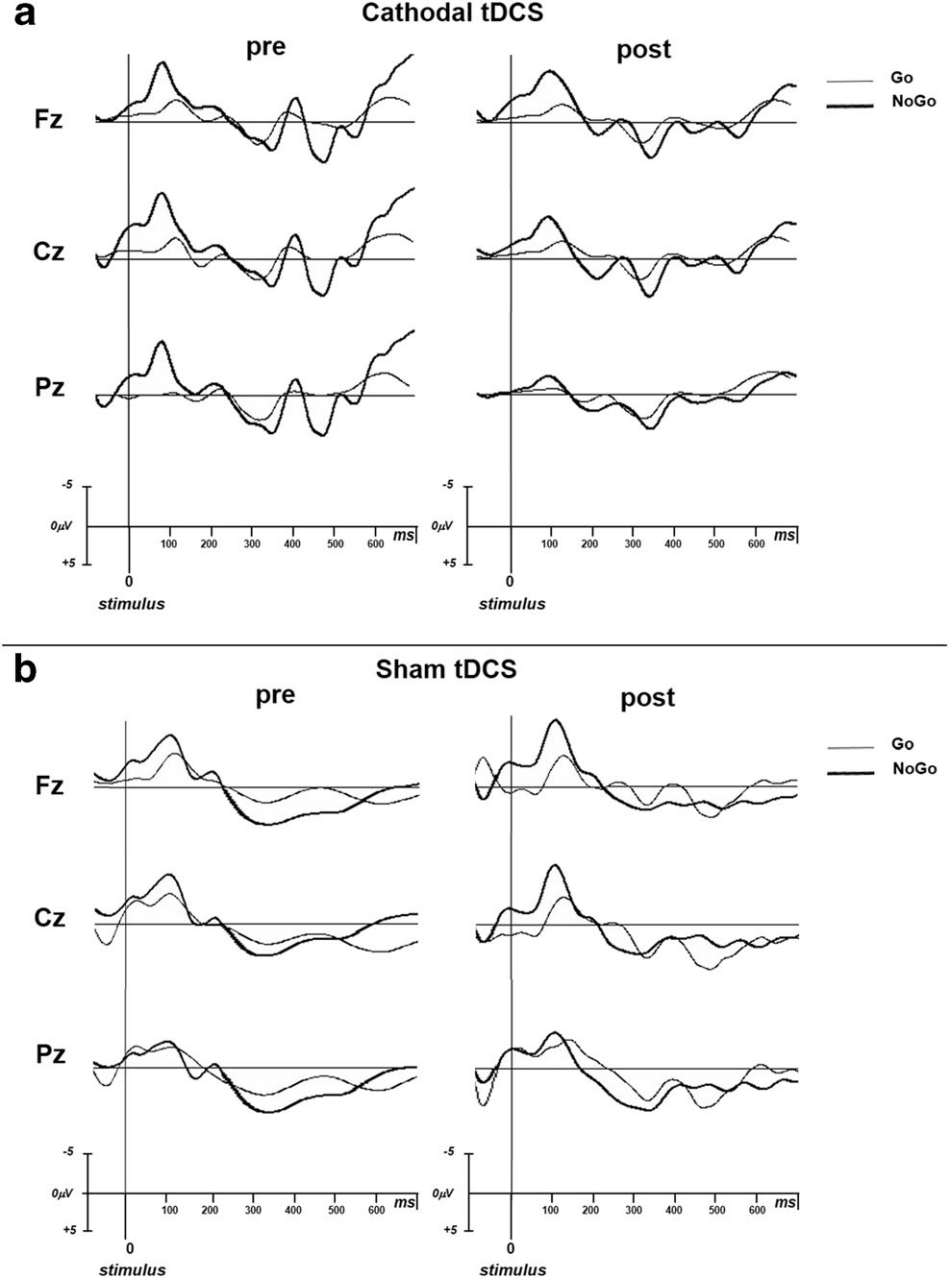
ERP traces in mid-line scalp locations for Go (thin line) and NoGo stimulus (thick line), both in pre-tDCS and post-tDCS, for cathodal (**a**) and sham (**b**) condition. The analysis epoch was 800 ms with a 100 ms pre-stimulus baseline before the stimulus

All subjects completed the two tDCS sessions without any complications. The subjects’ self-reported ratings of attention, fatigue, and perceived pain were not significantly different prior to and after tDCS stimulation across the two sessions (Table 1). For each session, the psychophysiological tasks were completed within 30 min of the cessation of tDCS. Figure 1 shows the grand average of the ERP components obtained during the Go/NoGo task.

**Table 1:** Psychological measures at pre-tDCS and post-tDCS evaluation

	Attention			Fatigue			Pain		
	Pre-tDCS	Post-tDCS	*p*	Pre-tDCS	Post-tDCS	p	Pre-tDCS	Post-tDCS	*p*
Cathodal	8.7 ± 1.4 (8.75)	8.7 ± 1.4 (9)	0.89	9.2 ± 0.9 (9)	9.0 ± 0.8 (9)	0.46	9.6 ± 0.7 (10)	9.6 ± 0.7 (10)	1.00
Sham	8.9 ± 1.2 (9.25)	8.7 ± 1.1 (9)	0.32	8.9 ± 1.2 (9)	8.8 ± 1.1 (9)	0.81	9.7 ± 0.5 (10)	9.4 ± 1.0 (10)	0.10

Values are displayed as mean ± SD (median) and depict the subject’s choice in a visual analogue scale in which 1 represents poorest attention, maximal fatigue, and pain, and 10 represents maximal attention, least fatigue, and pain. Significance level was set at *p* ≤ 0.05

## Go/NoGo Task

### N2

#### Amplitude

**Fig. 2:**
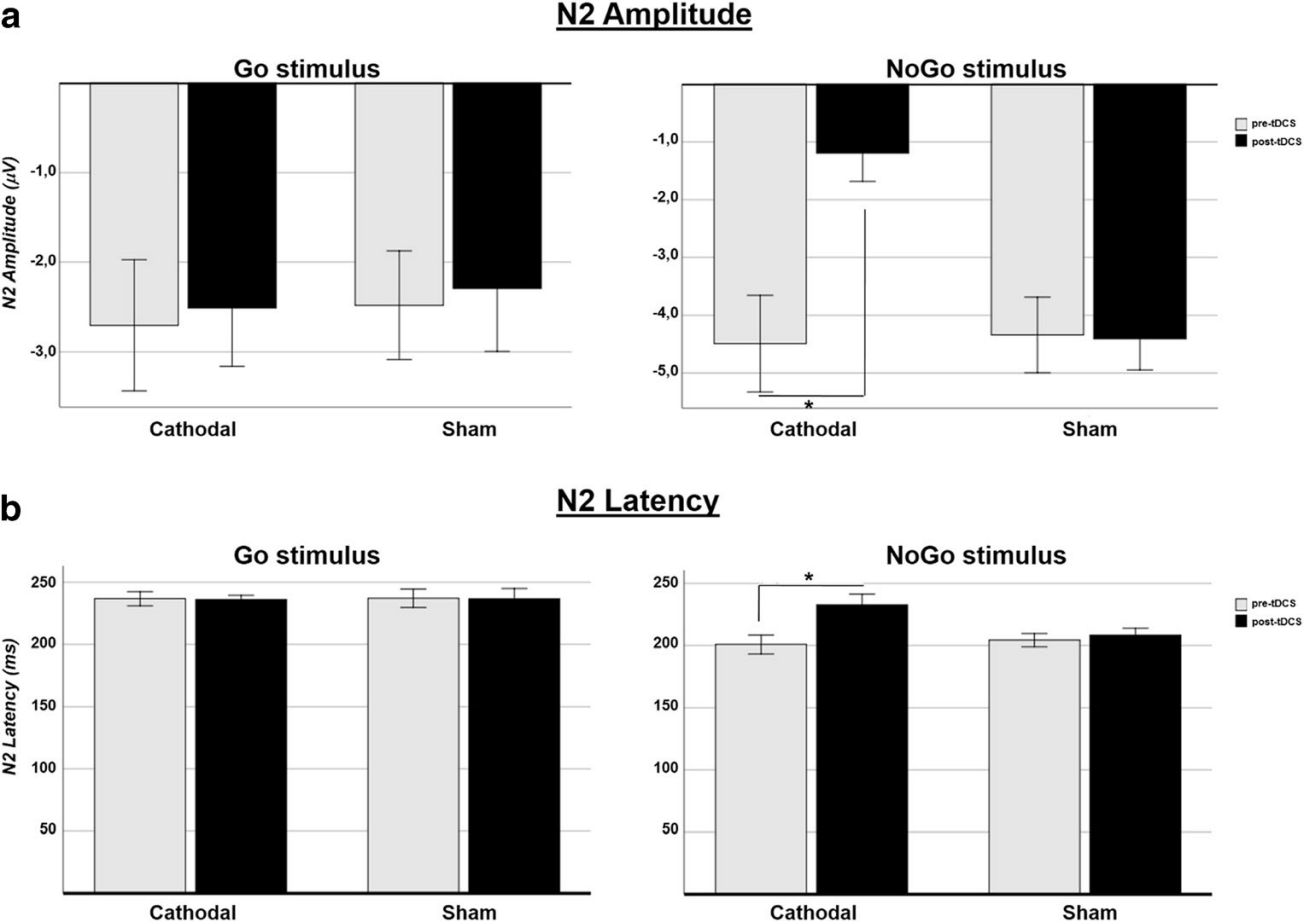
Amplitudes (**a**) and latencies (**b**) of N2 components for Go and NoGo stimulus are presented separately for cathodal and sham condition, both in pre-tDCS and post-tDCS. Error bars indicate ±1 SE. *p < 0.05

RmANOVA revealed a main effect of the “stimulus” factor (*F*_1–15_ = 3.78; *p* = 0.071; *η*^2^_p_ = 0.20), with higher amplitudes for the NoGo stimulus, of the “timing” factor (*F*_1–15_ = 6.06; *p* = 0.026; *η*^2^_p_ = 0.29), with higher amplitudes prior to stimulation, as well as of the “condition” factor (*F*_1–15_ = 4.55; *p* = 0.050; *η*^2^_p_ = 0.23), with higher amplitudes for the sham stimulation. Moreover, a significant “condition” × “timing” × “stimulus” interaction emerged (*F*_1–15_ = 8.46, *p* = 0.011, *η*^2^_p_ = 0.36); after Bonferroni’s correction, a significant difference emerged for the NoGo stimulus alone, with significantly lower amplitudes only after cathodal stimulation (pre, − 4.49 μV; post, − 1.19 μV; *p* < 0.001). By contrast, no difference emerged for either the Go stimulus (*p* = 0.82) or after the sham condition (Go *p* = 0.85; NoGo *p* = 0.91). Significantly lower NoGo-N2 amplitudes were detected after cathodal stimulation in frontal, central, and parietal sites (Fz: *p* = 0.001; Cz: *p* = 0.021; Pz: *p* < 0.001) (Fig. 2a).

#### Latency

RmANOVA revealed a main effect of the “stimulus” factor alone (*F*_1–15_ = 25.37; *p* < 0.001; *η*^2^_p_ = 0.63), which yielded, as expected, shorter latency amplitudes for the NoGo stimulus; no significant effect emerged for the “timing” (*F*_1–15_ = 3.07; *p* = 0.10; *η*^2^_p_ = 0.17), “condition” (*F*_1–15_ = 1.77; *p* = 0.20; *η*^2^_p_ = 0.10), or “electrode” (*F*_2–30_ = 0.07; *p* = 0.93; *η*^2^_p_ = 0.005) factors.

A significant “condition” × “timing” × “stimulus” interaction emerged (*F*_1–15_ = 5.23, *p* = 0.037, *η*^2^_p_ = 0.26). As observed for amplitude, after Bonferroni’s correction, a significant difference was detected for the NoGo stimulus alone, with significantly longer latencies after cathodal stimulation (pre = 200.8 ms; post = 232.6 ms; *p* = 0.003). By contrast, no difference was detected either for the Go stimulus (*p* = 0.93) or after the sham intervention (Go *p* = 0.94; NoGo *p* = 0.59). Significantly longer NoGo-N2 latencies after cathodal stimulation were detected in frontal, central, and parietal sites (Fz: *p* = 0.003; Cz: *p* = 0.003; Pz: *p* = 0.18). (Fig. 2b).

### P3

#### Amplitude

RmANOVA revealed a main effect of both the “stimulus” factor (*F*_1–15_ = 5.54; *p* = 0.033; *η*^2^_p_ = 0.27), with higher amplitudes for the NoGo stimulus, and the “electrode” factor (*F*_2–30_ = 19.1; *p* < 0.001; *η*^2^_p_ = 0.56), with higher amplitudes for centro-parietal than for frontal sites. No significant effect emerged for either the “timing” or the “condition” factors.

The “condition” × “timing” × “stimulus” interaction was not significant (*F*_1–15_ = 0.076, *p* = 0.78, *η*^2^_p_ = 0.005).

#### Latency

RmANOVA revealed a main effect of the “stimulus” factor alone (*F*_1–15_ = 3.60; *p* = 0.077; *η*^2^_p_ = 0.19), which yielded, as expected, longer latencies for the NoGo stimulus; no significant effect emerged for the “timing,” “condition,” or “electrode” factors.

The “condition” × “timing” × “stimulus” interaction was not significant (*F*_1–15_ = 0.044, *p* = 0.83, *η*^2^_p_ = 0.003).

### Performance Measures

#### Omissions (Go Stimulus)

Friedman’s test did not reveal a significant difference between omission errors measured pre- and post-tDCS stimulation (*χ*^2^ = 0.12; *p* = 0.99).

#### False Alarms (NoGo Stimulus)

**Fig. 3:**
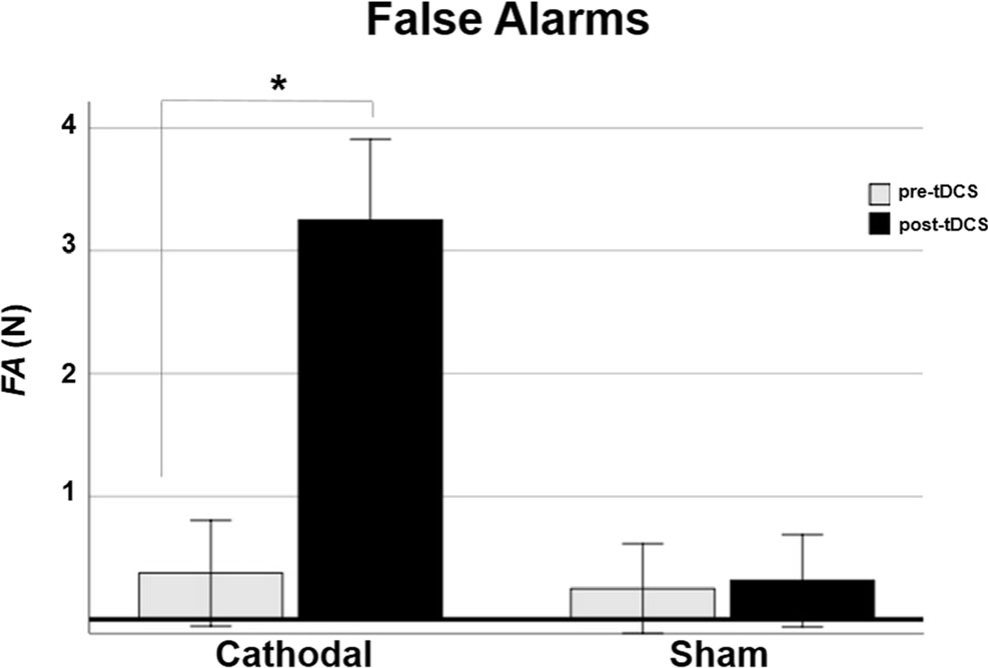
False alarms are presented separately for cathodal and sham condition, both in pre-tDCS and post-tDCS. Error bars indicate ±1 SE. *p < 0.05

Friedman’s test showed a significant difference between false alarm errors measured pre- and post-tDCS stimulation (*χ*^2^ = 34.72; *p* < 0.001); post hoc test using a Wilcoxon signed ranking test with Bonferroni-adjusted alpha level of 0.008 (0.05/6) showed that only after cathodal stimulation, the number of FA significantly increased (pre = 0.37; post = 3.25; *z* = − 3.6; *p* < 0.001). No differences were detected in FA after sham stimulation (pre = 0.25; post = 0.31; *z* = − 0.14; *p* = 0.89) (see Fig. 3).

#### Reaction Time

ANOVA did not reveal a main effect of either the “condition” (*F*_1–15_ = 0.154; *p* = 0.70; *η*^2^_p_ = 0.010) or “timing” (*F*_1–15_ = 0.034; *p* = 0.857; *η*^2^_p_ = 0.002) factors. Moreover, no significant “timing” × “condition” interaction was observed (*F*_1–15_ = 1.29; *p* = 0.27; *η*^2^_p_ = 0.08).

### SRT Task

#### P3

RmANOVA revealed a main effect of the “electrode” factor on P3 amplitude (*F*_2–30_ = 13.25, *p* < 0.001, *η*^2^_p_ = 0.47), which yielded, as expected, higher amplitudes in parietal sites than fronto-central sites. No other significant effect was observed (P3 amplitude: rest of Fs, *F* < 0.18; rest of ps, *p* > 0.69; P3 latency: all Fs, *F* < 0.497; all ps, *p* > 0.6).

#### Performance Measures

As regards reaction times, rmANOVA did not reveal a main effect of the “condition” (*F*_1–15_ = 0.124; *p* = 0.73; *η*^2^_p_ = 0.008) or “timing” (*F*_1–15_ = 1.49; *p* = 0.24; *η*^2^_p_ = 0.091) factors and no significant “timing” × “condition” interaction was observed (*F*_1–15_ = 0.449; *p* = 0.51; *η*^2^_p_ = 0.029).

As regards omission errors, Friedman’s test did not reveal a significant difference between errors committed pre- and post-tDCS stimulation (*χ*^2^ = 0.48; *p* = 0.92).

## Discussion

The aim of the present study was to investigate the role of the cerebellum in executive attentional functioning, in particular in inhibitory control, by evaluating the effects of cerebellar tDCS on the ERP components evoked during a Go/NoGo task.

Our data suggest that the processing of the NoGo stimulus alone is affected by cerebellar inhibition. Indeed, after cathodal tDCS, the latency of the N2-NoGo component was selectively longer than that observed at the basal evaluation. These data indicate that the orienting and initial discrimination of the incongruous stimulus are delayed. Moreover, the significant decrease in N2-NoGo amplitude also points to a reduction in attentional resources after cerebellar inhibition. Cerebellar inhibition does not instead seem to affect closure of the cognitive processing of the NoGo stimulus, as is suggested by the stability of P3-NoGo parameters before and after cathodal stimulation.

By contrast, processing of the Go stimulus does not appear to be affected by cerebellar inhibition in any of its phases, as indicated by the stability of both the N2 and P3 components before and after cathodal tDCS.

The Go stimulus is a stimulus that holds target information related to a motor act. Unlike the Go stimulus, the NoGo stimulus, which informs the subject to withhold the motor response, is by definition an incongruent stimulus and is thus able to activate on orienting response [60, 61]. In this regard, the selective alterations observed in the N2-NoGo latency and amplitude suggest that cerebellar inhibition in this study determined a selective dysfunction in the shifting of attentional resources toward an incongruent stimulus, which in turn resulted in an inefficient orienting response.

The orienting response toward an incongruent stimulus usually involves frontal-parietal brain areas in the ventral attention network, which identifies salient changes in the environment and acts as an alert system or circuit-breaker for the dorsal attention system [62], the latter of which directs attention to the target stimuli related to a specific task. The brain areas believed to be involved most in the generation of an orienting response are the cingulate cortices, the lateral prefrontal cortex, and the inferior parietal cortex [63]. Indeed, combined EEG-fMRI studies have shown that the medium cingulate cortex and the anterior cingulate cortex are prevalently activated during tasks related to the processing of incongruent stimuli or errors [63–65]. Furthermore, the lateral prefrontal cortex has been implicated in the maintenance and updating of information and in top-down control, as well as in association with the ACC in the monitoring of stimuli containing conflict information [66–69].

The aforementioned areas present dense connections, through the thalamus, with subcortical structures, particularly with the basal ganglia and the cerebellum [70]. The cerebellum is believed to coordinate the functioning of activities in these brain areas by regulating the level of activation or inhibition and managing the timing, speed, and appropriateness of cognitive processes [46]. In particular, it has been demonstrated that cerebellar output is conveyed mainly by an inhibitory pathway (the cerebellar-brain inhibitory tone—CBI) [46] that projects from the cerebellar cortex to the dentate nucleus and, from there, to the brain cortical areas through the thalamus [17, 71, 72].

By inhibiting this physiological inhibitory control, cathodal stimulation may have resulted in these cortical areas becoming hyperactive and uncoordinated [17, 44]. The non-synchronous and uncoordinated activation of areas involved in the perception of incongruous stimuli alters the orienting response specifically toward this kind of stimulus, which in turn elicits the psychophysiological components with a reduced amplitude. Indeed, the synchronized firing of cortical neurons activated by a selective cognitive task is known to be required to elicit psychophysiological components of an adequate amplitude [73–76].

This observation is in line with previous studies conducted by our group: a reduction in novelty-P3 amplitude was first observed after cerebellar cathodal stimulation [44]; moreover, an experiment in which ANT and tDCS were combined also highlighted the role of the cerebellum in executive attentional control specifically related to the processing of incongruous stimuli [17].

However, in a Go/NoGo task, the incongruous stimulus does not simply activate an orienting response. Within a Go/NoGo paradigm, the subject, who is unaware of which stimulus will occur in the sequence, is constantly alerted by the stimulus arrival so as to allow a prompt motor response to the Go-stimulus. The NoGo stimulus inhibits the preparation and execution of this response. Thus, it is likely that the altered NoGo-N2 parameters reflect a difficulty in inhibitory motor control. This finding appears to be supported by the increased number of false alarms, which are by definition errors linked to a failure to inhibit the motor response after the NoGo stimulus. By contrast, the discrimination of the incongruent stimulus is performed adequately (as demonstrated by the preserved NoGo-P3 amplitude and latency).

The cerebellum is reported to be directly involved in the processing of incongruent stimuli and in the inhibitory control of movement since it is able to perform specific sensory predictions according to the anticipatory or feed-forward model [77, 78]. Sensory predictions generated by a feed-forward model can be used to coordinate a motor act [79, 80], thereby providing a tool that plays a crucial role in anticipating the effects of a motor act and updating the motor system. These predictions may be compared with upcoming stimuli. If the predicted signals and the actual signals match, the afferent signals might not be considered whenever the system is updated. On the contrary, any difference that emerges between these stimuli constitutes a sensory prediction of the mismatch. As these signals of incongruity allow rapid adjustments in motor output, they may be considered indispensable to sensorimotor control. They are also crucial for learning, to refine future sensory predictions and to reduce the appearance of error on subsequent movements. The role of the cerebellum is likely to be related to the evaluation of temporal patterns of the stimulus as well as of those between stimuli and the related motor response [77]. Specifically, in a Go/NoGo task the cerebellum may help to generate a temporal contingency between the Go target stimulus and a motor response. When a NoGo stimulus appears, the role of the cerebellum is likely to exploit these conflict signals to improve future predictions and produce online changes in behavior related to incongruent information.

However, altered inhibitory control may even derive indirectly from a functional alteration of the basal ganglia. The basal ganglia include the subthalamic nucleus, which is connected both to the cerebellar cortex and to the deep cerebellar nuclei, and appears to act as the main gate for inhibitory control by regulating the functioning of supplementary motor areas [70, 81–83]. At the same time, the cerebellum seems to influence the functioning of the basal ganglia, and in particular of the indirect pathway, by modulating the activity of the striatal neurons connected with the external globus pallidus [84–86]. We believe that cathodal cerebellar inhibition alters inhibitory control by affecting the monitoring carried out by the basal ganglia of the programming, execution, and control of movements.

In summary, the executive attention dysfunction observed in our experiment, which is characterized by altered orienting to the incongruous stimulus associated with a reduction in inhibitory control, may derive in part from a direct cerebellar dysfunction and in part from an indirect dysfunction of the basal ganglia following cerebellar inhibition.

Our results do not seem to be in line with those of a previous study of Nozari et al. (2014) [87], which shows a facilitating effect for cognitive tasks after cathodal tDCS in the case in which the subjects were engaged in a minimally demanding task during stimulation. However, the authors used a different target of stimulation (prefrontal cortex instead of cerebellum) and a different cognitive task. In this perspective, these studies are not fully comparable.

To date, it is quite clear that the effects of tDCS can depend on several factors such as the applied tDCS intensity, duration, and timing stimulation [38–42] but also on task characteristics, the site of application, and the excitability status of the underlying cortical tissue [88]. This makes it rather difficult to find certain and repeatable effects of tDCS above all in cognitive studies [88, 89].

Given the relatively low focality of tDCS, we cannot rule out that neuronal activity in the right anterior cerebellum could be inhibited as well during cathodal session. In this sense, the higher false alarm rate following cathodal tDCS could be partly explain also with the inhibition of the motor response control. However, if the effect we found was purely motor, a variation of behavioral parameters would emerge also for the Go stimuli or during the simple reaction task. On the contrary, the reaction times to Go stimuli and the omission errors do not vary across sessions nor do the behavioral performances obtained during the simple reaction task. We believe that the effects induced by cerebellar cathodal inhibition are purely cognitive rather than related to motor-type effects.

To conclude, our experiment suggests that the cerebellum is involved in executive attentional functioning. In particular, the cerebellum seems to regulate attentional orienting to the stimulus and inhibitory control both directly, by making predictions of errors and behaviors related to errors, and indirectly, by controlling the functioning of the cerebral cortical areas involved in the perception of conflict signals and of the basal ganglia involved in the inhibitory control of movement.
